# The accuracy and effectiveness of self-gating signals in free-breathing 3D cardiac cine MRI

**DOI:** 10.1186/1532-429X-18-S1-P10

**Published:** 2016-01-27

**Authors:** Shuo Li, Yanchun Zhu, Jie Yang, Yaoqin Xie, Song Gao

**Affiliations:** 1 Shenzhen Institutes of Advanced Technology, Chinese Academy of Sciences, Shenzhen, China; 2 grid.11135.370000000122569319Peking University Health Science Center, Beijing, China

## Background

Conventional cardiac cine magnetic resonance imaging (MRI) is typically based on the segmented data acquired by breath-hold 2D balanced steady-state free procession (SSFP) pulse sequence [1]. However, the slice misregistration caused by the variation of breath holding positions could compromise the effectiveness of 2D SSFP, and breath-hold makes it difficult for pediatric patients or patients who suffer from congestive heart failure to cooperate [2]. Recently, respiratory and cardiac self-gated free-breathing (FB) cardiac cine MRI have been proposed for overcoming these barriers [3]. This approach could provide similar image quality and functional measurements without using electrocardiographic (ECG) or any peripheral equipment compared with 2D SSFP. However, the precision comparison between the self-gating signals and the external signals needs to be studied systematically.

## Methods

**Figure 1:**
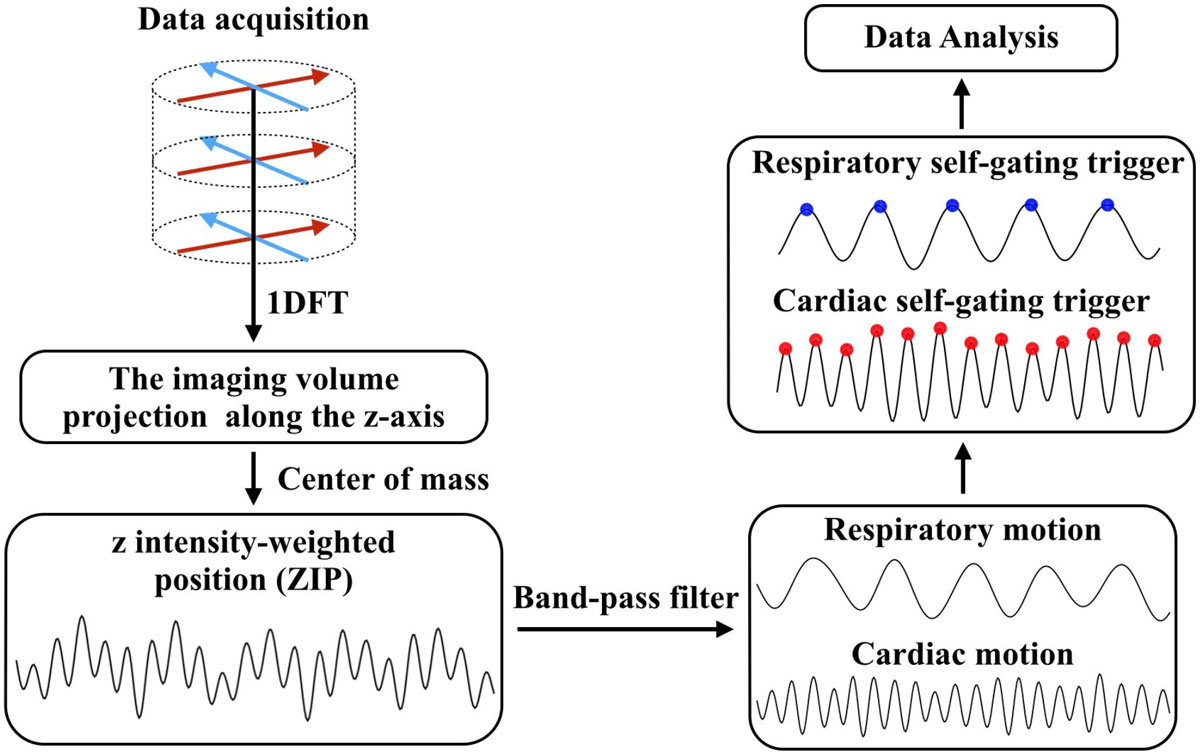
Figure 1

A self-gated FB 3D SSFP cine pulse sequence with hybrid radial *k*-space sampling based on golden angle was used for extracting the cardiac self-gating (CSG) and respiratory self-gating (RSG) signals. The peak positions of CSG/RSG signal curves were regarded as the CSG/RSG triggers. Respiratory bellows (RB) triggers and ECG triggers recorded by the peripheral equipment were used as the external triggers for comparison. The flow chart of this study is shown in Figure 1. The cardiac imaging was performed in 13 healthy volunteers on a 1.5T GE HDx scanner (maximum gradient amplitude 33 mT/m, slew-rate 120 mT/m/s, Excite 14M5 software; GE Healthcare, Waukesha, WI, USA). The study was approved by the local institutional review board and written informed consent was obtained from all subjects prior to enrolment. An eight-channel cardiac phased-array coil was used for signal reception. The cine imaging parameters were set as follows: TR/TE = 3.5/1.3 ms, flip angle = 40°, BW = ± 125 kHz, slice thickness = 7 mm (no gap), number of slices = 12, number of profiles = 5000, temporal resolution = 42 ms, total acquisition time = 4.5 minutes.

## Results

The mean heart rates are 81.302 (ECG) and 81.304 (CSG) bpm (beats per minute). The mean breathing rates are 21.934 (RB) and 21.933 (RSG) bpm (breaths per minute). The CSG and RSG cycle durations comparison, the correlation coefficient and the P value of two-tailed t-test are presented in Table 1. The RMSD between cardiac and respiratory cycle durations are 36.6 ± 8.8 ms and 328.1 ± 117.4 ms. The trigger time delay are 486.6 ± 78.2 ms (cardiac) and -309.0 ± 181.1 ms (respiratory).

**Table 1:** The cardiac and respiratory cycle duration obtained with peripheral equipment and self-gated FB SSFP technique.

	Cardiac cycle duration (ms)	Respiratory cycle duration (ms)
Peripheral equipment	750.5 ± 98.0	2748.7 ± 201.5
Self-gated FB SSFP	750.5 ± 98.1	2748.5 ± 200.1
Correlation (p = 0)	0.60 ± 0.14	0.64 ± 0.13
T-test P value (H=0)	0.98 ± 0.02	0.98 ± 0.02

## Conclusions

Self-gating signals can well synchronize the cardiac and respiratory motion. It has excellent correlations with the external signals and the RMSD is acceptable. The t-test shows that there is no significant difference between two methods. Future study will focus on the comparison of self-gating signals extraction algorithms.

## References

[1] Hudsmith LE (2005). J Cardiovasc Magn R.

[2] Sievers B (2011). Acta Cardiol.

[3] Liu J (2010). Magn Reson Med.

